# Functional foods: promising therapeutics for Nigerian Children with sickle cell diseases

**DOI:** 10.1016/j.heliyon.2022.e09630

**Published:** 2022-06-02

**Authors:** Oladeji John Alabi, Fikayo Noah Adegboyega, Dolapo Samuel Olawoyin, Oluwakemi Arinola Babatunde

**Affiliations:** a Department of Biochemistry, Faculty of Basic Medical Sciences, Ladoke Akintola University of Technology, Ogbomoso, Nigeria; b Department of Biochemistry, Institute for Agriculture & Natural Sciences, College of Arts & Sciences, University of Nebraska-Lincoln, USA; c Department of Biotechnology, Egypt-Japan University of Science and Technology, Alexandria, Egypt; d School of Food Science and Nutrition, University of Leeds, Leeds, UK; e Department of Nursing, Achievers University, Owo, Nigeria

**Keywords:** Functional foods, Sickle cell disease (SCD), Pediatric/children

## Abstract

Sickle cell disease (SCD), also known as sickle cell anemia (SCA) is one of the structural hemoglobinopathies that occurs due to a single nucleotide mutation from GAG to GTG, which changes the amino acid of a β-globin chain of hemoglobin (Hb) from glutamate to valine. This singular mutation results to disorderliness in red blood cells (RBCs) with advent of changes in RBC morphology and other pathological conditions. In the 1980s, intermittent red blood cell transfusions, opioids, and penicillin prophylaxis were the only available therapy for SCA and were commonly reserved for acute, life threatening complications. So far, the US Food and Drug Administration (FDA) has granted a total of four drugs approval for the prophylaxis and treatment of the clinical complications of SCD. Due to limitations (adherence, safety, adverse effects) of existing therapies in the prophylaxis and treatment of SCD complications in Nigerian children and their inaccessibility to approved drugs, the present study discusses the therapeutic effects of readily available functional food as one of the therapies or an adjunct therapy to tackle the sickle cell crisis in Nigerian Children.

## 1 Introduction

Sickle cell disease (SCD) is a group of genetic ailments affecting the red blood cell [1]. It is sometimes referred to as drepanocytosis consisting of sickle thalassemia and sickle cell anemia (HbSS). Heterozygous genotypes (HbAS) expressing parents are sickle cell carriers and their offspring have 25 % chances of giving rise to a homozygous sickle genotype (HbSS) progenies or a 75 % homozygous normal genotype (HbAA) [2]. Sickle cell derangement was first identified as a hematological disease by Herrick in 1910 [2] and its biochemical pathology was proven by Linus Pauling in 1949 [2]. Epidemiological information on SCD has majorly been reported among Jamaica, India and US population, with less statistics in Africa where the trait of SCD is said to be largest [3]. It is mostly common among the black Africa, and some other races occupying the Mediterranean, India, and Middle East [4]. Further studies on children born with SCD have recorded the major occurrence in the developing world, with an estimated 200,000 annual sickle genotype births in Sub-Saharan Africa [5].

Sickle cell disease is one of the causes of childhood mortality in Africa. 80% of people with SCD globally live in Sub-Saharan Africa. This proportion is projected to reach 88% by 2050 [6,7]. Nigeria has the largest burden of sickle cell disease in Africa and the world, with approximately 150,000 births annually [8] and 50–90% of them die before age five [9]. Poor quality healthcare, poor education, unawareness, poverty, and poor nutritional choices contribute to excess mortality. In West African for example, historical and scientific records have described different names used to qualify SCD children among the three main Nigerian tribes. The Yorubas, constituting the dominant population in the west called them “abiku” translated “sufferers'' or “children that bring sadness” [10, 11], the Ibos called them “Ogbanje” [12], and the Hausas called them “Sankara-miji” [13]. It is the major innate disorder in Nigeria, affecting about 4 million population at prevalence of 2% at birth while more than 40 million persons have sickle cell traits.

Nigeria shows a record for about 75% of children with SCD in Africa [14]. Though having normal weight at birth, babies affected by SCD show weight loss during the first year and gradually lingers until adulthood incidentally followed by prolonged skeletal maturation in both boys and girls with a protracted menarche in girls [15]. At molecular and genetic levels, the hereditary sickle cell derangement is visualized when a single nucleotide base substitution occurs in the gene coding human β-globin subunit. Such substitution causes the replacement of hydrophilic amino acid (glutamic acid) at position 6 in normal hemoglobin (HbA) by a hydrophobic amino acid (valine) in abnormal hemoglobin, culminating in the disease state of sickle red cells [16]. The occurred hydrophobic replacement results in insolubility of sickle cell hemoglobin (HbS) when deoxygenated. HbS molecules that are constantly formed polymerize to long crystalline intracellular mass of fibers that deform the original biconcave shape of the red blood cell (erythrocyte) into a sickled shape cell. The magnitudes of this reshaping are noticed with hemolytic anemia and tissue disruption emanated from the blockage of blood vessels by the sickled cells.

Clinical appearances of this condition are vascular necrosis, hyposthenuria, proliferative retinopathy, priapism, aplastic crises, pulmonary disease and nephropathy. In most cases, the complications are severed and include periodic attacks of pain and progressive organ dysfunction leading to a much reduced biologic life span [17]. Likewise, reactive oxygen species [18] are considered to play a crucial role in the SCD pathogenesis. The chronically increased oxidative stress in SCD might play a significant role in the emergence of SCD related organ complications [19].

Pharmacologically, many drugs with various targeted pathways have emerged. However, majority of them have failed to display benefit in medical trials, few have produced encouraging results but with less availability making them out of reach for low – income countries like Nigeria [20]. According to WHO (2002), up to 80% of individuals living in Africa depend on traditional plant-based treatment, with slight side effects, for their primary health care needs [18]. Phytomedicine has been widely utilized as effective remedies for the prevention and treatment of multiple health conditions for centuries by almost every known culture in Nigeria [18].

Polyphenols, alkaloids, flavonoids, and other Phenolic compounds have been reported [21] to have antisickling and antioxidant effects which are capable of restoring normalcy to the titled redox balance that originally contributes to sickle cell crisis in children. Such foods containing polyphenols are often referred to as functional foods i.e. foods containing bioactive compounds that can promote the health of a person beyond rudimentary diets, or serve as a measure for prevention or management of chronic diseases [22].

Nevertheless, the application of renowned drugs and potential curative treatments will most likely be limited to high-income countries who are able to possess them at high cost, leaving out the low – income countries with severe complications due to unavailability of these therapeutics (Table 1). In this review, we discuss the readily available unique functional foods that can be used in ameliorating the sickle cell crisis in Nigerian Children.

**Table 1:** Clinical trials of the FDA approved pharmaceutical drugs for sickle cell disease.

Clinical Trials	Mechanisms and Results	Recommended Dosage & Cost	Side Effects
**Hydroxyurea** Multicenter Study of Hydroxyurea (MSH)NCT00000586 [54] Multicentre, randomised, controlled trial (BABY HUG) NCT00006400 [55]	**Targeting HbS polymerization:** Inhibits Ribonucleotide reductase; thereby increasing HBF levels, which in turn retard sickling. Drug was well tolerated. Reduction in the need for blood transfusions and frequency of painful episodes, lower hospitalization rates, and reduced number of episodes of acute chest syndrome in children and adults.	Start with 15 mg/kg/day and then gradually increase the dose by 5 mg/kg/day every 4- to 8-weeks until it reaches the maximum tolerated dose (MTD) which is 30–35 mg/kg/day. ICER estimated the annual costs of Hydroxyurea to be $1,200.	Thinning hair (mild hair loss), dermatologic changes (skin hyperpigmentation or dark fingernail beds), and nausea. There are chances for HU to increase embryofetal toxicity, low sperm count, and myelosuppression [77, 78].
**L-glutamine** Phase III randomized trial NCT01179217 [69]	**Targeting hemolysis-mediated endothelial dysfunction:** Increase NADH and NAD redox potential; thereby maintaining vascular tone & impairing adhesion. Drug was well tolerated; reduction in the number of pain crises, lower hospitalization rates, and reduced number of episodes of acute chest syndrome in children and adults with SCD with or without hydroxyurea compared to those that received placebo. No improvement in Hb or reticulocyte count.	0.3 g/kg glutamine powder is administered orally twice per day. The estimated cost is above $3000/month for adults and $1000/month for children, which is 20 times more expensive than hydroxyurea [65]. The estimated annual cost is $24,000 [ICER].	Nausea, headache, constipation, cough, and abdominal pain
**Crizanlizumab** Phase II randomized trial NCT01895361 [70]	**Targeting vasocclusion:** Humanized monoclonal antibody against P-selectin; thereby reducing pain crises. All SCD genotypes experienced tremendous reduction in the percentage of crisis episodes by 45% in high-dose arm; no improvement in markers of hemolysis.	5 mg/kg is administered intravenously over a period of 30 min at weeks 0, and 2, followed by 5 mg/kg once every 4 weeks. 100mg cost $2828.6 while the annual costs is estimated to be $88,000 [ICER].	Nausea, arthralgia, back pain, and pyrexia
**Voxelotor (Oxbryta/GBT440)** 1/2, RDBPC, NCT02285088/NCT03041909 [74] Phase III randomized trial NCT03036813 [75]	**Targeting HbS polymerization:** Binds to HbS a-globin chain and stabilizes it in the R-state; thereby increasing its affinity for oxygen. Voxelotor was well tolerated. Significant improvement in hemoglobin levels and markers of hemolysis.	1500mg once daily. Voxelotor is available as a 500-mg oral tablet, and its estimated cost is $138.89 per tablet [76] while its annual cost is $84,000 [ICER].	Headache, diarrhea, nausea, and arthralgia

## 2 Pathophysiology of Sickle Cell Disease

**Figure 1:**
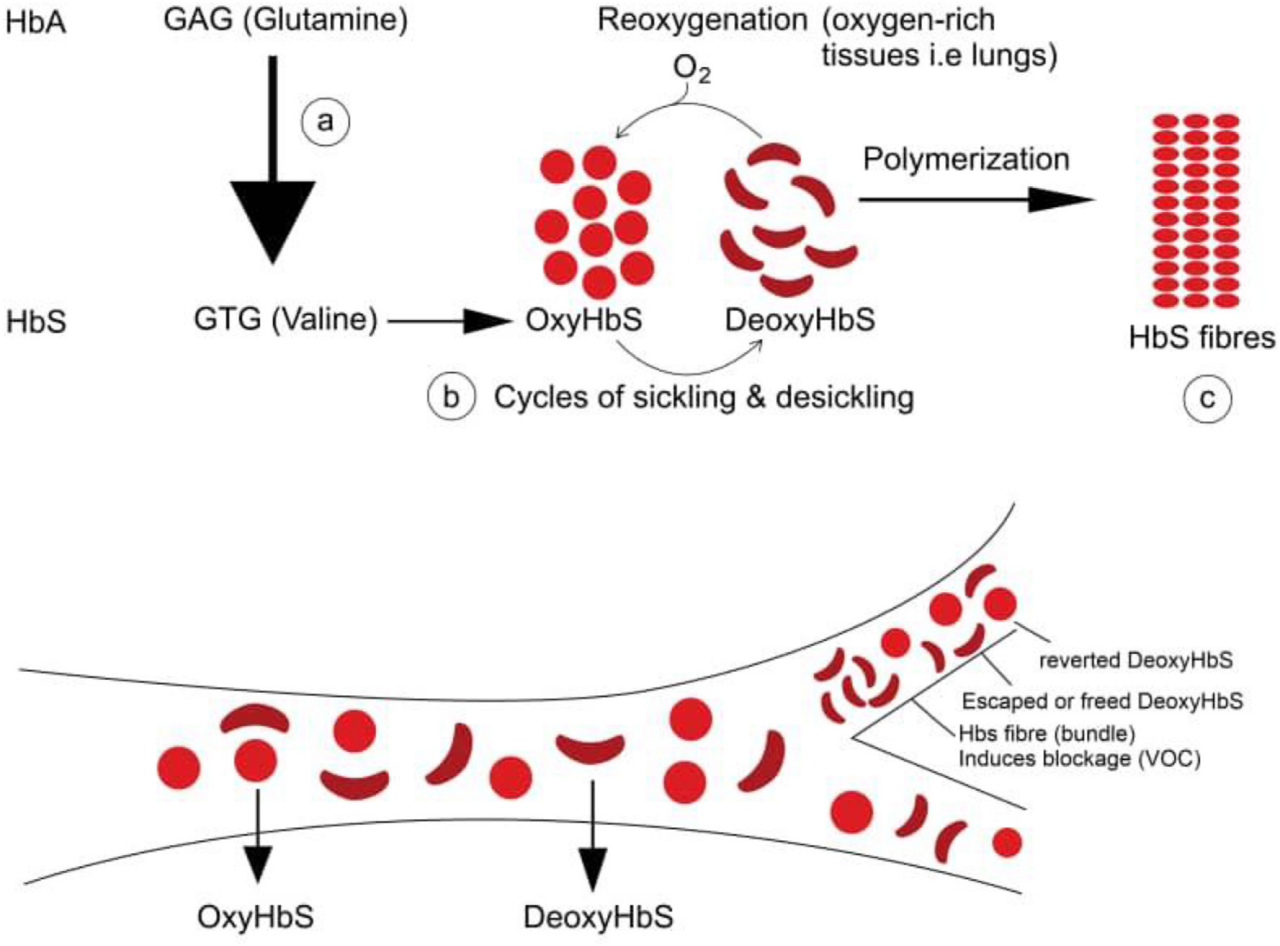
Schematic of RBC sickling in the vasculature: (a) point mutation from GAG to GTG cause a normal adult hemoglobin HbA to become sickle HbS (b) OxyHbS takes a sickle or banana shape after deoxygenation and polymerizes to form (3) HbS fiber, leading to vaso-occlusion. However, some HbS molecules, with the sickle shape (DeoxyHbS) manage to escape polymerization and undergo reoxygenation and revert back to OxyHbS (desickling). Multiple cycles of sickling and desickling predisposes HbS molecules to hemolysis leading to hemolytic anemia [1, 26].

**Figure 2:**
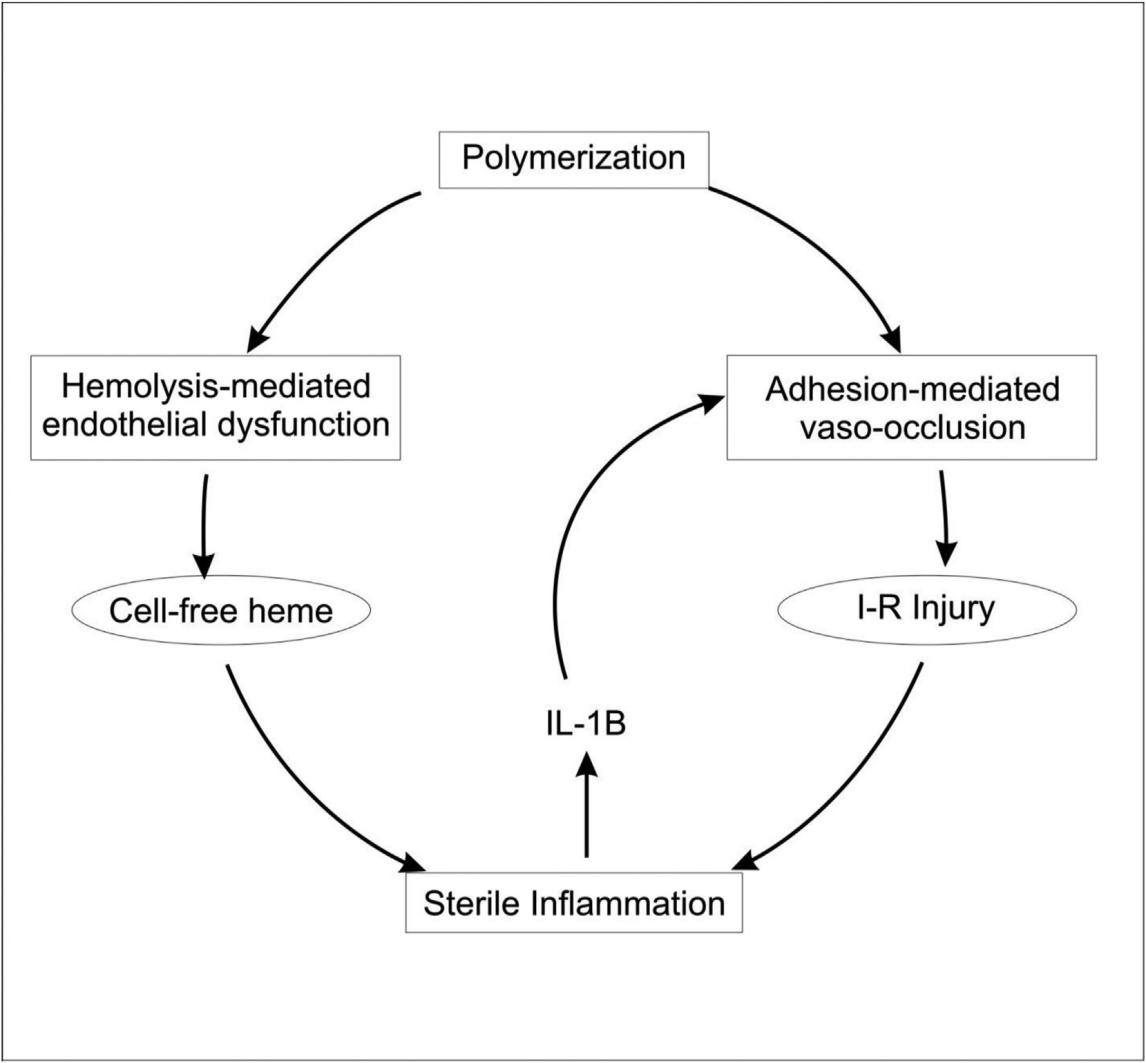
Pathophysiology of sickle cell disease. Adapted from [24].

Sickle cell disease (SCD), also known as sickle cell anemia (SCA) is one of the structural hemoglobinopathies that occurs due to a single nucleotide mutation from GAG to GTG, which changes the amino acid of a Beta-globin chain of Hb from glutamate (hydrophilic) to valine (hydrophobic). This mutation is a point mutation, and it occurs at the 6th position or codon on the Beta-globin chain [23]. This single action produces an abnormal globin chain and is responsible for the unique properties of a sickle cell (Figure 1). The pathophysiology of sickle cell anemia entails four different parts occurring in a vicious cycle: (*1*) polymerization, (*2*) hemolysis, (*3*) vaso-occlusion, and (*4*) sterile inflammation (Figure 2) [24,25].

### 2.1 Polymerization

When the red blood cell (RBC) of a sickle patient becomes deoxygenated, it presents a sticky patch on its surface and tends to stick to other hemoglobin molecules to form a polymer of mutated sickle cell hemoglobin (HbS) molecules. The polymers then aggregate to form an insoluble, long chain of rigid fiber (bundle) that distort and modify the RBC membrane resulting in sickling. Piccin and colleagues [27] reported that valine hydrophobicity is responsible for this sickling because it attracts hydrophobic regions of adjacent β-chains during polymerization. This sickling property (Figure 1) is the trademark that leads to the downstream of events -hemolysis, vaso-occlusion, and sterile inflammation, including the manifestations of a sickle RBC.

### 2.2 Hemolysis

During oxygenation and deoxygenation, the sickled red blood cells become fragile and prone to rupture (hemolysis) probably due to loss of their membrane filaments leading to anemia. This portrays the short lifespan of 10–20 days seen in a sickle cell as opposed to the 100–120 days in a normal RBC. The premature destruction of HbS RBC (hemolysis) has been shown to mediate endothelial dysfunction by releasing cell-free hemoglobin molecules into the blood. The hemoglobin molecules in their oxygenated state of Fe2+ oxidize to form methemoglobin (Fe3+), a non–oxygen-binding form of hemoglobin with inert nitrate (NO3−) and hydroxyl free radical (OH·) as shown in the reaction given below.

1) Fe2+ + NO ➝ NO3− + Fe3+ deoxygenation reaction

2) Fe2+ + H202 ➝ OH· + Fe3+ Fenton reaction

In the equation one above, the cell-free Hb scavenges and transforms NO into nitrate. NO impairs a myriad of adhesion molecules and maintains platelets in steady state, thus preventing endothelial damage. Hence, depletion of NO by free Hb would invariably promote endothelial dysfunction. In addition, in the process of hemolysis, arginase is being released intravascularly from the destructed sickle red blood cells. Arginase reduces nitric oxide (NO) bioavailability by consuming plasma arginine levels (the latter being an important substrate for nitric oxide (NO) synthases), thereby, aggravating NO depletion and further damaging the endothelial NO-sustained mechanisms of vasodilation [27, 28].

Endothelial enzymes ➝ O2·– + NO ➝ ONOO-

Similarly, endothelial enzymes such as xanthine oxidase, uncoupled endothelial NO synthase, and NADPH oxidase that are produced via catalysis by hemoglobin and heme including damaged tissues, neutrophils, and platelets also contribute to the endothelial dysfunction through the production of superoxide anion radical (O2·–) that depletes endothelial NO to produce peroxynitrite (ONOO-). Several studies [28, 29] have shown that these ROS originates from numerous sources, and sickle RBCs have an impaired ability to neutralize them due to their reduction of endogenous antioxidants. Subsequently, methemoglobin (Fe3+) breaks down to release cell-free heme giving rise to erythrocyte damage-associated molecular pattern (eDAMP). The eDAMP destructs redox balance of the cell, and promotes oxidative stress and sterile inflammation.

### 2.3 Vaso-occlusion

Past and current studies have indicated the interplay among HbS polymerization, impaired blood rheology, hemolysis, and increased adhesiveness of sickle RBCs with inflammatory cells and vascular endothelium, and vaso-occlusion activation. However, the cellular and molecular mechanism of action of vaso-occlusion still remains uncertain. Also, evidence has shown that blood vessels occlusion can be initiated by diverse mechanisms involving different inflammatory and/or environmental stimuli such as TNFα, hypoxia, heme, dehydration, Hb, infection, hypoxia, acidosis, lipopolysaccharide, and several others [30, 31, 32, 33, 34]. It has been reported that these stimuli or triggers may cause pain events due to a link or connection between the vaso-occlusion and autonomic nervous system [35]. Further, it has been explained that the cellular and molecular paradigm of vaso-occlusion is not similar in all vascular beds or organs [24].

After deoxygenation, the HbS bundle takes a sickle or needle-like shape that protrudes through the lipid bilayer to cause a leakage [36], which causes the influx of calcium ions inside the HbS. This action activates the HbS RBC ion channel (Ca2-dependent K channel, K–Cl cotransport) resulting in a corresponding efflux of potassium ion and water out of RBC [27, 37]. This further distorts the RBC and makes the RBC more viscous and dehydrated. The HbS polymer bundles also stimulate membrane lipids peroxidation to cause the release of peroxidation products which can damage the membrane structure, alter water permeability, and increase cell deformability. Disruption of the RBC membrane phospholipids causes the exposure of phosphatidylserine (PS) to the outer cell surface [38]. This loss in RBC integrity makes it detectable and markable by macrophages and spleen for destruction, thereby elevating the level of hemolysis, and thus contributing to chronic anemia. Most destruction of sickle hemoglobin takes place extracellularly, while only a fraction occurs intravascularly and make up 30% of total hemolysis [36]. During hemolysis, heme is produced. Hemolysis, heme, and dehydration have been indicated to propagate or contribute to vaso-occlusion. In an attempt to compensate for blood shortage during or following hemolysis, the bone marrow produces more reticulocytes than normal, therefore, it becomes stressed and produces immature reticulocytes, known as “stress reticulocytes” with adhesion molecules- α4β1 integrin (VLA-4) and CD36 expressed on their surface [1, 31, 39].

### 2.4 Sterile inflammation

The sterile inflammation pathway has been indicated to be activated by a concerted action of both hemolysis-mediated endothelial dysfunction and increased adhesion-mediated vaso-occlusion [24, 40, 41] (Figure 2). Either of the cell-free heme or ischemia-reperfusion (I-R) injury promotes sterile inflammation in SCA by activating the inflammasome pathways in vascular and inflammatory cells to release Interleukin-1β (IL-1β). The released or activated IL-1β aggravates the progression of vaso-occlussion by promoting the adhesiveness of platelets, neutrophils, and endothelial cells, upregulation of P-selectin, E-selectin, intercellular adhesion molecule-1 (ICAM-1), vascular cell adhesion molecule-1 (VCAM-1), and chemokines [28, 42]. In the process of inflammasome activation, heme and I-R injury have been shown to be involved in the generation of reactive oxygen species (ROS), neutrophil extracellular trap (NET), generations of DAMPs, and DNA, and activation of the Toll-like receptor 4 (TLR4) [40,43,44,45,46,47]. In other words, ROS, NET, TLR4, DAMPs, and DNA play a critical role in Inflammasomes activation following their stimulation and/or generation by heme or I-R injury. Therefore, deletion/inhibition of any of these molecules might prevent or attenuate sterile inflammation in SCD.

## 3 FDA approved pharmaceutical drugs for sickle cell disease in clinical practice

So far, the US 10.13039/100009210Food and Drug Administration (10.13039/100000038FDA) has granted a total of four drugs approval —Hydroxyurea, L-glutamine, voxelotor, and crizanlizumab—for the prophylaxis and treatment of the clinical complications of 10.13039/501100016391SCD. In the 1980s, intermittent red blood cell transfusions were the only available therapy for SCA and were commonly reserved for acute, life threatening complications [48]. Blood transfusions were claimed to be associated with a number of risks, including acute transfusion reactions, transmission of infection, the development of allo- and auto-antibodies to erythrocyte and human leukocyte antigens, and transfusional hemosiderosis (iron overload) leading to further damage to organs such as the heart, pancreas, and liver [49, 50]. In addition, there are other supportive therapies such as hydration and opioids analgesics that are commonly used along with blood transfusion to manage the painful events of sickle cell disease.

### 3.1 Hydroxyurea (hydroxycarbamide)

In the 1990s, hydroxyurea (HU) emerged as a promising pharmacologic therapy for SCA [48] and was approved for use in adults with SCD by the US Food and Drug Administration in 1998 [29]. Further, hydroxyurea received US FDA approval for the treatment of children (pediatric patients) from 2 years of age and older with severe SCA in 2017. The clinical studies of hydroxyurea pluripotency to induce fetal hemoglobin (HbF) in sickle cell anemia started in the mid- 1980s [51,52], followed by the phase I/II [53], phase III/MSH [54], and BABY HUG [55] trials which led to its approval in adults first- 1998 and then children-2017 (Table 1). These approvals were granted to reduce the frequency of painful crises and the need for blood transfusions in both children and adults of SCA with recurrent moderate to severe painful crises.

**Figure 3:**
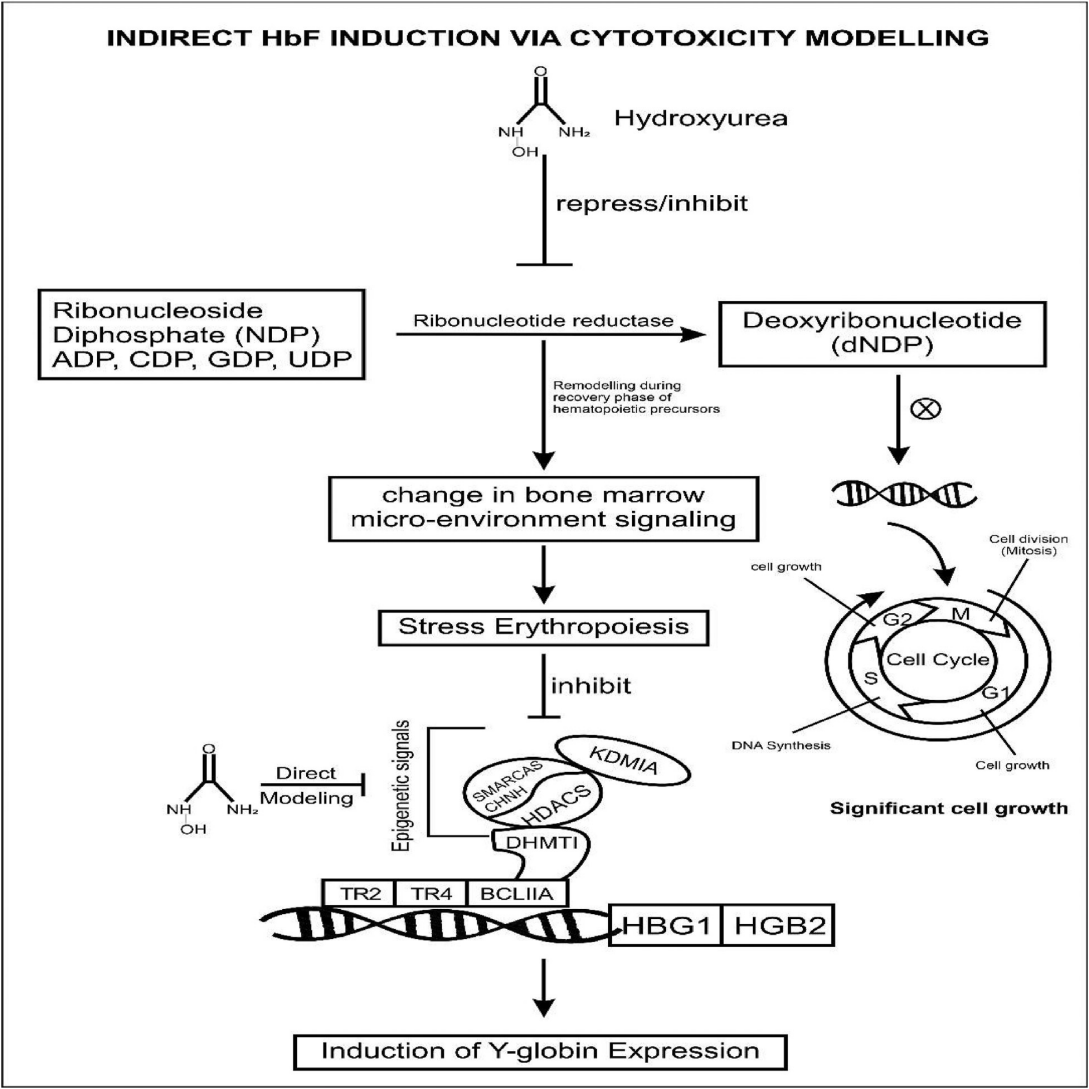
**HU Mechanism of Action via the Epigenetic and Post Transcriptional Modification:** HU inhibits ribonucleotide reductase, the enzyme that converts ribonucleosides (NDP) into deoxyribonucleosides (dNDP). dNDP is required for the synthesis and repair of DNA. Therefore, the inhibition of this enzyme impedes the progression of cellular division (DNA synthesis) through the S-phase, thereby altering erythroid kinetics and causing a reversal via a recovery phase of hematopoietic precursors. This indirect remodeling enhances the recruitment of early erythroid progenitors (such as stress erythropoiesis), which silence the epigenetic signals/enzymes and induce Y-globin expression. HU can also remodel Y-globin gene loci by directly repressing the epigenetic enzymes. DNA methyltransferase 1 (DNMT1); various histone deacetylases (HDAC), lysine demethylase 1 (LSD1, KDM1A); chromodomain helicase DNA binding protein 4 (CHD4); SWI/SNF-related matrix-associated actin-dependent regulator of chromatin subfamily A member 5 (SMARCA5); DNA-binding factors DRED (TR2/TR4); BCL11A: B-cell CLL/lymphoma 11A (BCL11A); Hemoglobin subunit gamma-1 & 2 (HBG1/HBG2) Adapted from [56].

**Figure 4:**
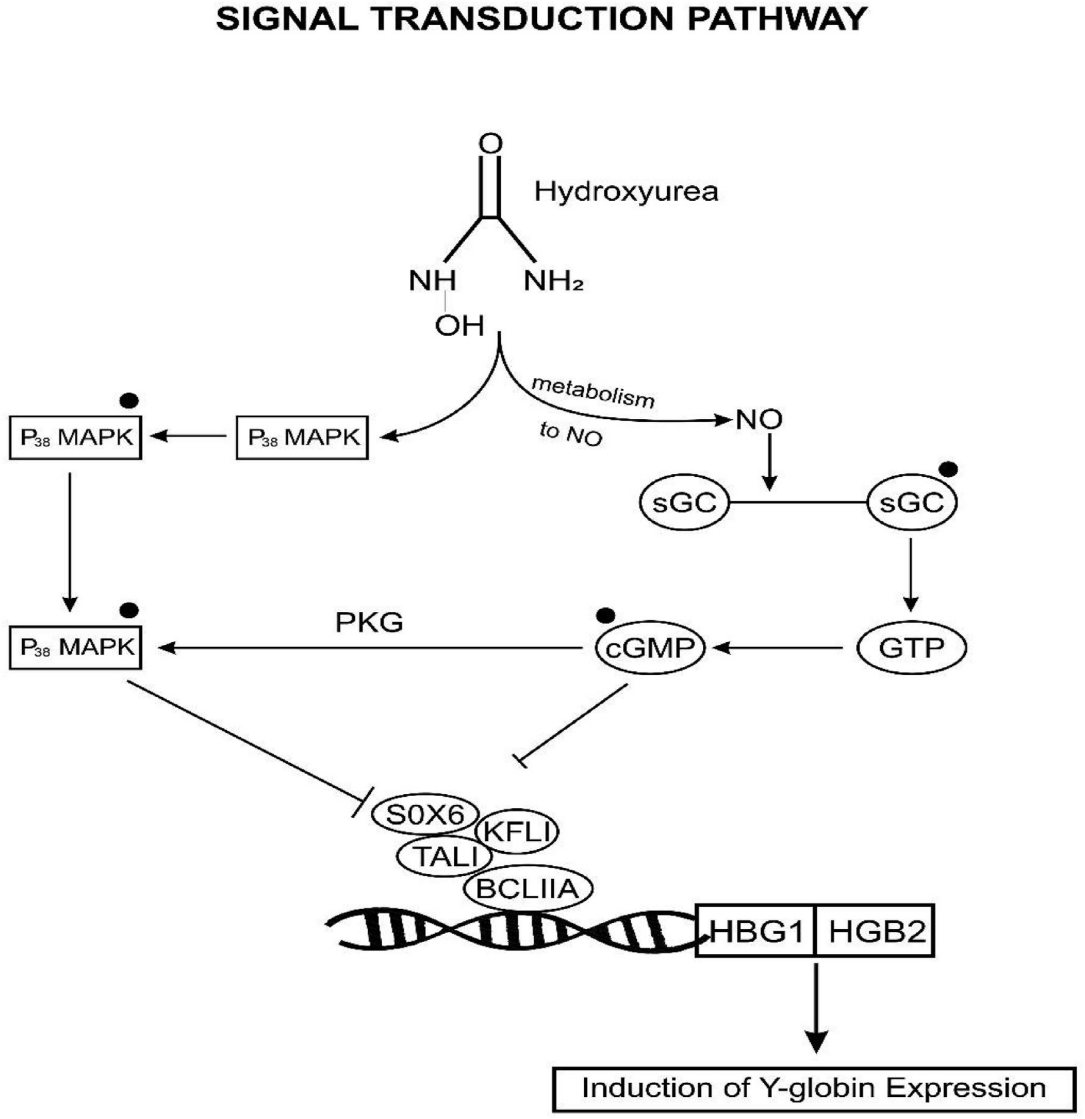
**Hu Mechanism of Action Via the Signaling Pathway:** NO: nitric oxide; sGC: soluble guanylyl cyclase; GTP: guanosine triphosphate; cGMP: cyclic guanosine monophosphate; p38 MAPK: p38 mitogen-activated protein kinase; SOX6: SRY-Box Transcription Factor 6; LF1: Kruppel Like Factor 1; T-ALL: T-cell acute lymphoblastic; BCL11A: B-cell CLL/lymphoma 11A. Adapted from [57].

The pharmacological interventions of hydroxyurea for SCA relate to its ability to increase fetal hemoglobin (HbF) levels. The HbF-containing γ-globin chain is the principal hemoglobin found during the pregnancy period of the individual. Six weeks after birth, the HbF is replaced by the adult hemoglobin containing the Beta-globin chain. Hydroxyurea adopts a mechanism of action that favors a continued production of the fetal hemoglobin instead of the adult hemoglobin whose Beta-globin gene becomes abnormal over time. Therefore, HU can elevate HbF levels, which in turn inhibits/retards HbS polymerization. Hydroxyurea has multiple mechanisms of action and profound benefits in responders [29]. Although, the exact mechanisms of action of hydroxyurea remain incompletely understood. However, some of these mechanisms of action have been elucidated [56, 57]. The most significant mechanism of action is the reversible inhibition of ribonucleotide reductase, followed by the signal transduction pathway (Figures 3 and 4). Several reviews have claimed that hydroxyurea might work by mechanisms beyond its effects on HbF and that its full benefits in SCA are multifactorial [48, 58]. Piccin and associates further summarized the seven most relevant modes of action of hydroxyurea [27]. Some of these possible mechanisms of action show significant impact on the erythrocyte cation transport (improvement in RBC hydration), sickle cell membrane, adhesion molecules (reduction of neutrophil count, leukocyte adhesion, and pro-inflammatory markers), NO generation, erythropoietin production, and red cell deformability [59, 60, 61, 62, 63, 64].

### 3.2 L-glutamine (Endari)

**Figure 5:**
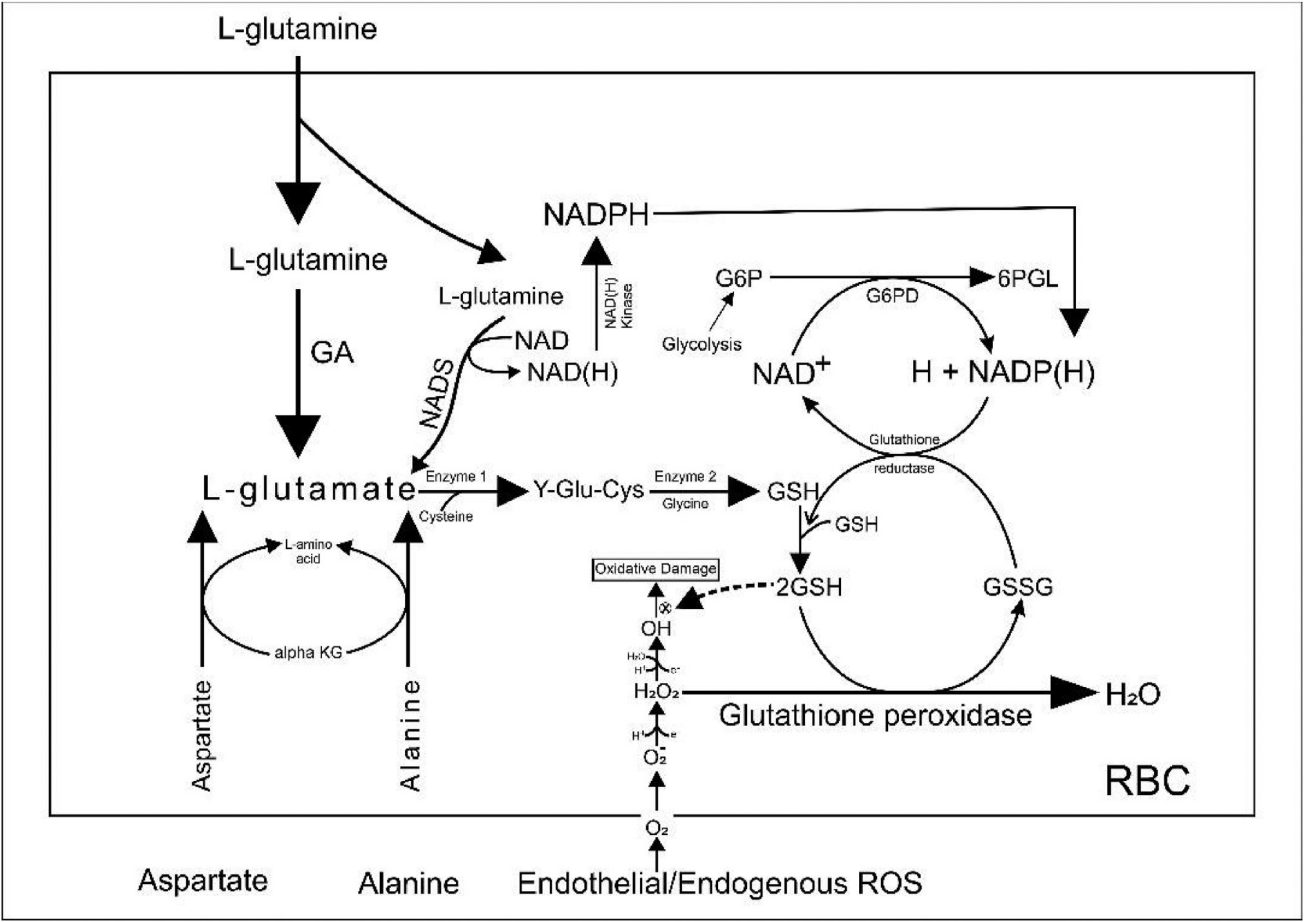
**Proposed Mechanism of Action for L-glutamine:***GA: glutamine aminohydrolase;* aKG: A-ketoglutarate; NADS: nicotinamide adenine dinucleotide synthase; G6P: Glucose 6-phosphate; G6PD: Glucose 6-phosphate dehydrogenase; Enzyme 1; Y-glutamyl cysteine synthetase; Enzyme 2: glutathione synthetase; 6-PGL: 6-Phosphogluconolactone; GSH: Reduced Glutathione; GSSH: Oxidized Glutathione. Adapted from [65].

The L-glutamine oral powder (Endari) is the second drug approved in July 2017 by the Food and Drug Administration for the treatment of complications of SCD in 5 years and older individuals [25, 49]. Several studies and extensive reviews have shown oxidative stress to be a critical factor in the pathophysiology of SCD. The mechanism of action of L-glutamine is not fully understood, although it has been associated with the protection against RBC oxidative damage (Figure 5). L-Glutamine is an amino acid and precursor required in the synthesis and production of glutamate (Glu), glutathione (GSH), arginine (Arg), and nicotinamide adenine dinucleotide (NADH), all of which protect the RBC from oxidative damage and indirectly maintain vascular tone [65]. NADH/NADPH is an oxidative reductant that plays a critical role in maintaining the redox potential of the cell. It helps to regenerate and recycle reduced glutathione (GSH) which is essential for the detoxification of hydroxyl radicals (OH·) and hydrogen peroxides (H_2_0_2_). The combined activities of NADPH and GSH help to mop up endogenous ROS, stabilize and protect the RBC against oxidative damage or stress. Additionally, the production of arginine plays a significant role in Nitric Oxide (NO) production thus, enhancing NO bioavailability and preventing endothelial dysfunction.

A study demonstrated by Morris and colleagues [66] in SCD patients showed a significant reduction in total glutathione and glutamine levels compared to normal healthy individuals. The depletion of glutamine levels was freely associated with pulmonary hypertension and contributed to RBC membrane damage and endothelial adhesion. Further, in the past and recent studies of Niihara and colleagues, L-glutamine therapy - administered orally - improved the redox potential of sickle RBC and showed a significant reduction in the endothelial adhesion of sickle cells to the human endothelial cells of the umbilical vein [67, 68]. In a phase 3 trial (Table 1), L-glutamine demonstrated a reduction in the number of pain crises, lower hospitalization rates, and reduced number of episodes of acute chest syndrome in children and adults with SCD with or without hydroxyurea compared to those that received placebo [69]. Nevertheless, none of these studies shows any improvements in Hb or reticulocyte count.

### 3.3 Crizanlizumab (Adakveo)

**Figure 6:**
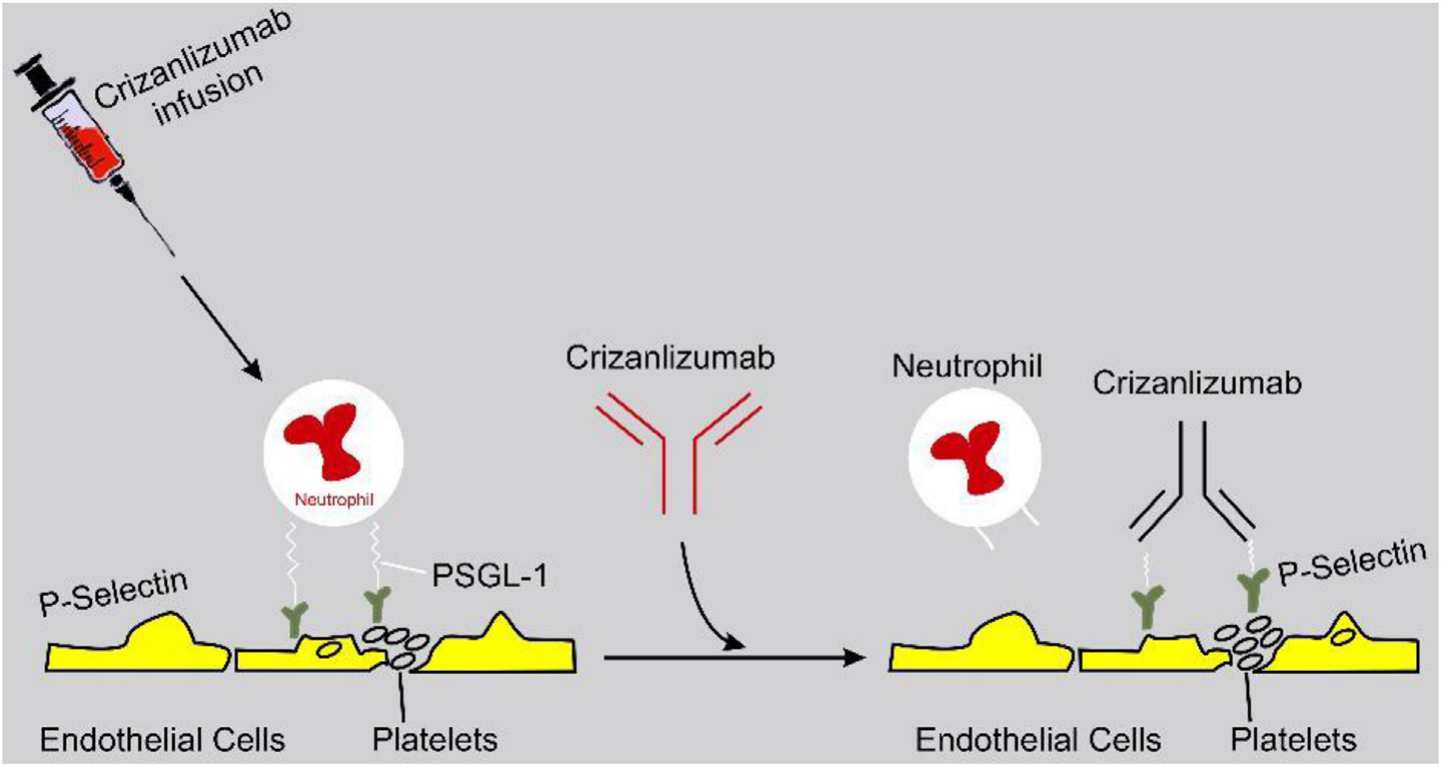
**Mechanism of action of Crizanlizumab.** P-selectin is predominantly expressed on the endothelial cells and platelets. Adapted from [https://www.creativebiolabs.net].

The US FDA approved crizanlizumab for sickle cell patients above 16 years of age in November 2019. Crizanlizumab is a humanized monoclonal antibody targeted against the vaso-occlusion pathway to attenuate vaso-occlusion crisis. It binds with P-selectin to inhibit P-selectin interaction with other adhesion molecules-neutrophils, platelets, and activated erythrocytes in the endothelial (Figure 6). In a recent study trial conducted by [70, 71], crizanlizumab demonstrated a significant reduction in the episodic crisis experienced by sickle cell patients compared with the placebo group with or without hydroxyurea.

### 3.4 Voxelotor (Oxbryta/GBT440)

Voxelotor was approved by the FDA in November 2019, for the treatment of SCD patients 12 years of age and older. Voxelotor is a hemoglobin modulator, thus increasing hemoglobin levels. It binds to the N- terminal valine of the alpha subunit of hemoglobin and increases its affinity for oxygen [72]. The increased affinity of the sickle cell hemoglobin for oxygen stabilizes the hemoglobin and prevents polymerization (sickling) [73]. Recent studies conducted during trials showed a concurrent rise in hemoglobin levels and a significant reduction in the markers of hemolysis (unconjugated bilirubin, percentage of reticulocytes, except LDH) and the percentage of sickled red cells when offered voxelotor (Table 1) [74, 75]. Voxelotor is generally well tolerated and safe with moderate adverse outcomes-headache, diarrhea, nausea, and arthralgia [76]. Patients who are hydroxyurea resistant with continuous hemolysis and anemia may consider a voxelotor supplement as an additional agent.

## 4 Nutrition and sickle cell disease

Nutrition plays a significant role in children with SCD; however its importance tends to be overruled due to the preference given to the clinical predictors of SCD morbidity including dactylitis prior to age 1, a haemoglobin <7 g/dL and leukocytosis [79]. In a steady state, a sickle cell patient is most likely to experience a significant reduction in energy intake and a significant increase in energy expenditure during crises. This is probably due to the increased in metabolic demands driven by protein and bone turnover caused by chronic hemolysis, resting metabolic rate, glucose and lipid fluxes, thus producing a negative energy balance and undernutrition [79, 80]. According to a study from Heyman and associates [81], it is easier to speculate *a priori* that the reason for low dietary and energy intake may be due to a loss of appetite from the early stage of life. Additionally, proinflammatory mediators-cytokines - are likely to be associated with hypophagia in both sickle cell children and adults during steady states [79].

12 weeks after birth, sickle children develop lower weight (wasting) and height (stunting), which persists through adolescence and adulthood. Both wasting and stunting are usually associated or linked with/to poor nutritional status, impaired growth and immune functions. Children with SCD have been indicated to manifest different degrees of macro- and micro-deficiencies, which is associated with SCD painful crises and the frequency of hospitalizations [82, 83]. Nutritional supplementation with polyunsaturated fatty acids, magnesium, iron, zinc, folate, protein, energy, vitamin A, C, D and E, have all been suggested with the great potential benefit of preventing the painful crises of SCD and promoting healthy growth in children with SCD [84]. Therefore, there is the need to consider the roles of different nutrients and integrate nutritional interventions in the management of children with SCD.

## 5 Functional foods and nutraceuticals as adjunct therapies in managing SCD

**Figure 7:**
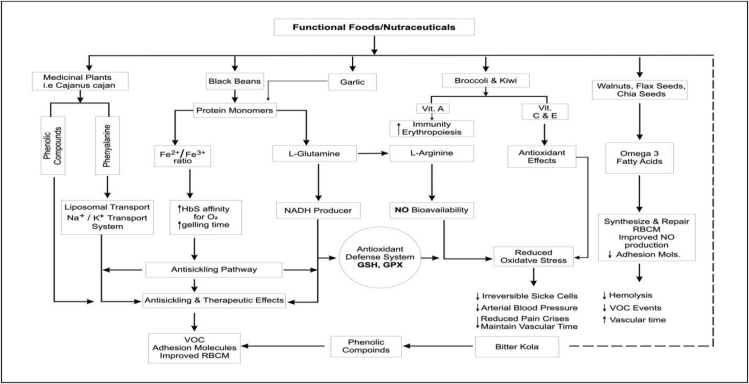
Graphical summary of actions of some functional foods.

The terms “Functional foods” and “Nutraceuticals” have become popular in the scientific world, and mostly adopted in twenty first century by the food and nutritional researchers who are currently working on how to solve various crises arising from the degenerative diseases, and some genetically triggering diseases like sickle cell diseases. The terms have been used in many reviews and articles with the aim to stress the importance of foods in mitigating diseases. At the molecular level, functional foods and nutraceuticals are said to bring about some interactions which are due to the bioactive components or ingredients present in these compounds (Figure 7 & Table 2). Managing SCD has created many gaps for recent research with many clinical trials trying to reduce the complications and pain episodes that may result from SCD.

**Table 2:** Main Functions of Functional Foods and their Correlations with FDA-Approved Drugs during Sickle Cell Crisis.

S/N	Functional Foods	Bioactive Compounds	FDA-Approved Pharmaceutical Drugs	Main Functions
1	Black Beans (*Phaseolus vulgaris L.)*	Amino acids [22]	L-glutamine Hydroxyurea Voxelotor	**Targeting HbS polymerization:** Antisickling effect, membrane stability, and Antioxidant potentials [21, 86]
2	Aged Garlic *(Allium sativum)*	Flavonoids, Organosulfur [108] and Amino acids [109]	L-glutamine	**Targeting hemolysis-mediated endothelial dysfunction:** Antioxidant potentials [104]
3	Bitter Kola *(Garania kola H.)*	Phenolic compounds such as: Tannins, Guttiferin, Flavonone, etc. [98]	Crizanlizumab	**Targeting vasocclusion:** modulate inflammatory responses (Membrane stability) [100]
4	Broccoli *(Brassica oleracea)*	Vitamin A [92]	Crizanlizumab	**Targeting vasocclusion:** Membrane stability and protective [99]
5	Kiwi *(Actinidia deliciosa)*	Vitamin C Vitamin E [92]	L-glutamine	**Targeting hemolysis-mediated endothelial dysfunction:** boost the glutathione antioxidant defense system thereby promoting Antisickling and therapeutic properties [92]
6	Walnuts *(Juglans regia);* Flax seeds *(Linum usitatissimum);* Chia seeds *(Salvia hispanica)*	Omega – 3 fatty acids [112, 113]	Crizanlizumab L-glutamine	**Targeting vasocclusion:** Anti-adhesive, anti-agreggatory and anti-inflammatory properties [114]

According to ((FAO), 2007), Functional food is any food with physiological benefits, and capable of reducing the risk of chronic disorder beyond the normal nutritional trait such food possesses. More so, it is said to be the food that provides and equips the body with wealthy health ((FAO), 2007). Nutraceuticals as explained by Ferrari [85] is also a food or part of foods that has clinical benefits, and capable of preventing/treating few or many disorders. Countries like Nigeria still face the increased rate of SCD crises in children, and the different strategies erected to manage the crises are narrowly unavailable for every individual due to the cost in procuring them (Table 1). This review elucidates the adoption of the functional foods and nutraceuticals as one of the therapies or adjuvants of treatment to manage SCD diseases in Nigerian children. This is because functional foods are readily available and occur naturally in edible medicinal plants’ seeds, fruits, leaves or their cooked/extracts.

### 5.1 Antisickling effects

**Figure 8:**
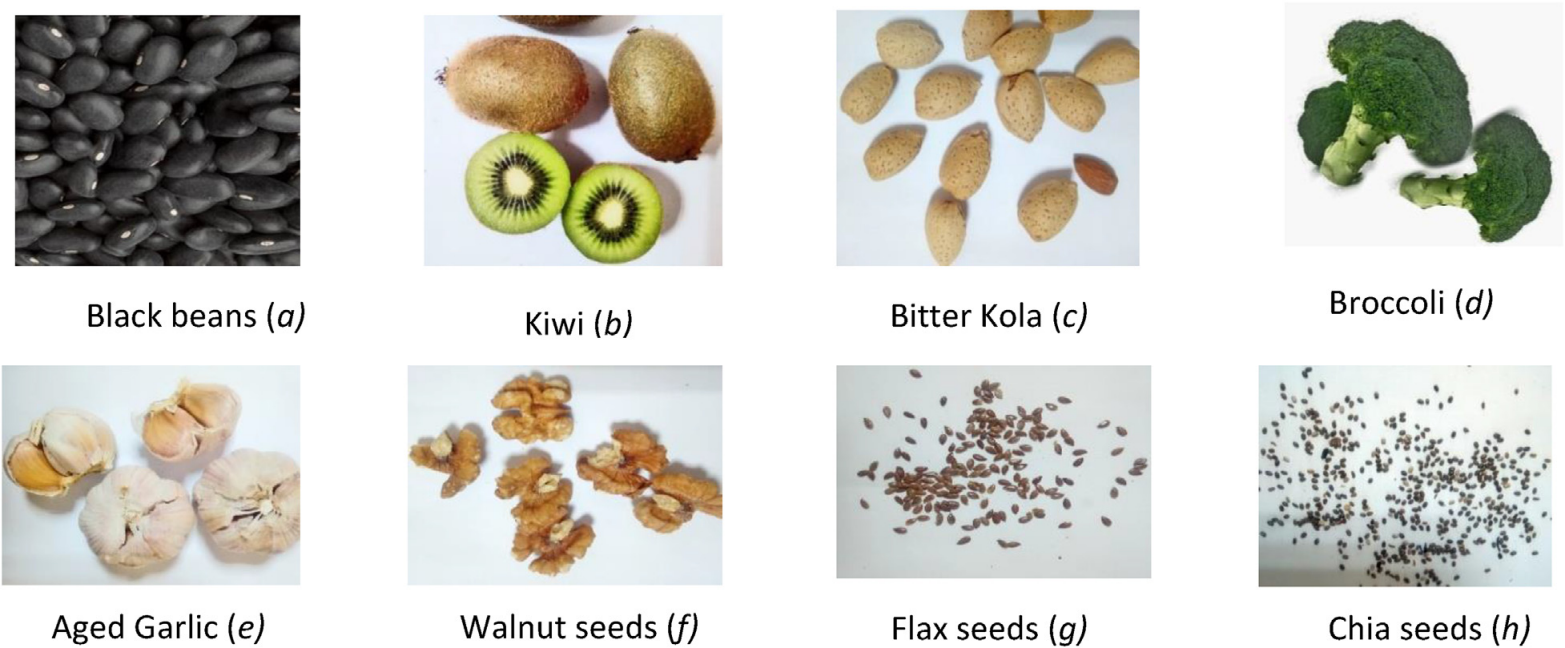
**Pictures of Functional Foods: (a)** Black beans (*Phaseolus vulgaris* L); **(b)** Kiwi (*Actinidia deliciosa*); **(c)** Bitter kola (Garania kola H.): **(d)** Broccoli (*Brassica oleracea*); **(e)** Aged garlic (*Allium sativum*); **(f)** Walnuts seeds (*Juglans regia*); **(g)** Flax seeds (*Linum usitatissimum*) **(h)** Chia seed (*Salvia hispanica*). (Listed functional foods were purchased from local market in Borg El Arab, Egypt).

Black beans are generally used as a source of plant proteins [86], and they come in a variety of seed shapes, sizes, and colors all over the world (Figure 8a). Black beans contain a total of 16 amino acids with a variety of health effects, according to HPLC profiling [87]. Bean products are found to include albumin, globulin, and phenolic chemicals, all of which have strong antioxidant characteristics [21, 86]. These chemicals have antisickling properties and can reduce oxidative stress, which is linked to the SCD crisis. According to a study, individuals in Cameroon's West Region frequently use black bean seed varieties to treat SCD [88]. The bioactive chemicals contained in bean products have been reported to protect against cardiovascular disease and cancer, and other chronic diseases in various studies [89, 90]. Their antioxidant activity and capacity to chelate metal catalysts are the reasons for this [21].

The reduction in the Fe2+/Fe3+ ratio, as previously stated, is one of the downstream events that occurs during polymerization in sickle cells. Polymerization causes ferrous iron (oxyhemoglobin-Fe2+) to oxidize to ferric iron (methemoglobin-Fe3+), preventing oxygen fixation. The antioxidant, antisickling, and membrane stability capabilities of black bean components have all been studied [87]. The findings reveal a rise in Fe2+/Fe3+ ratio, DPPH/OH∗ scavenging effect, and a considerable reduction in HbS polymerization and hemolysis, all of which are related to the distinct roles of both L-glutamine and hydroxyurea. The presence of antioxidant amino acids in black beans may explain why they can inhibit ferrous iron oxidation and so boost sickle cell hemoglobin's affinity for oxygen in individuals with SCD, similar to how voxelotor works. Furthermore, some of these amino acids' polar/hydrophobic character is thought to have assisted their capacity to diffuse into the HbS molecule and bind at the heme pocket, preventing the protein-protein interaction required for gelation [91]. These observations suggest that black beans could be a promising anti-sickling, membrane stability, and antioxidant agent in the treatment of SCD.

Another functional food with antisickling properties is kiwi fruit (Figure 8b). Kiwi is rich in both vitamin C and E [92]. Dietary supplementation with Kiwi as given by [92], maybe an important adjunct during SCD therapy. Antioxidant vitamins C and E can boost the glutathione antioxidant defense system and exhibit an inhibitory role in red blood polymerization [2]. Its combination with anti-SCD therapeutic drugs has been reported to reduce the incidence and severity of sickle cell crisis [93]. In the findings of Kawchak and colleagues [94], functional foods containing vitamins E and C have the propensity to improve the health conditions of SCD children. The effects of these vitamins have been shown to decrease arterial blood pressure, mean corpuscular hemoglobin levels, and irreversible sicklde cells [2]. Vegetable and fruit diets such as kiwi may contain vitamin C and E [92] with a great antisickling property and therapeutic benefits in sickle cell anemia management cases [95].

### 5.2 Membrane stability

Bitter kola is a medicinal seed with an abundance of biologically active products (Figure 8c). An ethnobiological survey has shown that some families in Oyo State, Nigeria [96] and in the Littoral region of Cameroon [88] are fond of using bitter kola for the management of SCD. Bitter kola seeds have been reported to contain several phenolic compounds such as guttiferin, tannins [97], garcinia flavanone, xanthenes, benzophenone, kolaflavanone, and biflavonoids [98]. These chemical constituents possess antibacterial, antiviral, and anti-inflammatory properties [99], which could prevent infections and modulate inflammatory responses in SCD. An investigative study on bitterkola seed confirmed that it was more effective in stabilizing erythrocyte membranes as seen/observed in HbAA, HbAS, and HbSS genotypes [100].

More so, antioxidant fruits and vitamins are protectors and maintainers of the membrane structure of RBCs, preventing them from being depleted by free moving radicals during SCD. Broccoli [91] (Figure 8d) is one of the functional foods that provide Vitamin A, an effective substance that improves iron absorption during RBCs formation and differentiation by increasing the mobilization of iron from the tissue stores. It also enhances erythropoiesis and immunity [101]. In the study of Boadu, intake of Vitamin A in children with SCD has significant importance and improves their health [102]. This result also supports the findings of [103].

### 5.3 Antioxidant potential

Aside antioxidant fruits, garlic has been declared a versatile vegetable with great antioxidant potentials [104] (Figure 8e). The bioactive compounds [105] in garlic are highly effective against peroxidation and free radicals that often cause damage to cells and organs. Aged garlic extract (AGE) has been proclaimed to be safer [106] and exerts potent antioxidant effects in vitro and in vivo compared to fresh garlic [93, 107]. It enhances antioxidants levels in the body (ascorbic acid, vitamin E, reduced glutathione (GSH), glutathione peroxidase (GPx), superoxide dismutase (SOD), and catalase). The flavonoid and the organosulfur component of garlic prevent blood clots and keep blood vessels fit [108]. According to a study by Nwaoguikpe [109], the proximate composition and phytochemical profile of garlic comprise 18 amino acids and vitamin C. Another study evaluated the antioxidant effect of AGE on RBCs via Heinz body analysis [110]. In the above studies, AGE inhibited the polymerization process, stabilized and improved the oxidant status of the erythrocyte membranes by improving the Fe2+/Fe3+ ratio and decreasing Heinz bodies. Similarly, AGE has been reported to inhibit dense cell formation (dense bodies) in vitro and ex vivo [111]. The high level of amino acids, S-allyl cysteine, and antioxidant vitamins identified in AGE must have contributed to the erythrocyte deformability and antisickling potency of garlic in the management of SCD.

### 5.4 Anti-adhesive, anti-aggregatory and anti-inflammatory properties

Walnuts, Flaxseeds, and Chia seeds (Figure 8f) may be another important dietary supplement for SCD crises. Omega – 3 fatty acids [112] present in walnuts, Flaxseeds, and Chia seeds [92] as reported by Daak and colleagues [113] play a significant role in improving the overall health of children with SCD by reducing vaso-occlusive crisis. This is probably due to its anti-adhesive, anti-aggregatory, vasodilatory and anti-inflammatory properties [114]. Synthesis and repair of the red blood cell membrane (RBCM) require omega – 3 and omega- 6 fatty acids which are collectively called polyunsaturated fatty acids (PUFAs). Lack of foods rich in PUFAs may cause defects in the repair system of the RBCM, thus retarding the neural maturation and development, and some other sensory systems. Recent studies have explained the role of sickle vasculopathy-vascular inflammation and thrombosis-in the generation of sickle cell complications [115, 116, 117]. Recently, sickle ce**ll** mice studies have demonstrated that supplementation with Omega – 3 fatty acid diet normalizes RBC membrane, improves NO production, reduces inflammation and adhesion molecules [118]. With the above features, one might hypothesize that Omega – 3 fatty acids expresses/possesses the unique activity of crizanlizumab and L-glutamine. Therefore the higher the level of Omega – 3 fatty acids in the blood, the lower the risk of complications or severe symptoms of SCD, and the lesser the degree of anemia [119].

### 5.5 Seed, leaf and root extracts of plants

Many medicinal roots, leaves, and seeds that contain beneficial phytochemical compounds have been utilized since the onset of SCD to reduce sickling and painful occurrences. Leaf extracts of *Carica papaya* and *Parquetina nigrescens*, root extracts of *Fagara zanthoxyloides*, and seed extracts of *Cajanus cajan* are antioxidant plant extracts, which also play vital roles as antisickling agents [2]. *In vitro* research on extracts of *Carica papaya* leaves has indicated its potency in reducing hemolysis and preserving erythrocyte membrane integrity [9, 120]. Similarly, the extract of *Parquetina nigrescens* exerts anti-sickling activities and confer protection on the integrity of the RBC membrane [121]. Another study conducted on a cocktail of *Carica papaya, Parquetina nigrescens*, and *Fagara zanthoxyloides* extracts shows promising results [122]. These pharmacological properties are due to the presence of several phenolic compounds and free amino acids found in these plant extracts [123, 124, 125]. *Eugenia caryophyllata* and *Piper guineense* are other examples of medicinal plants thatcontain vanilloids like Shikimic acid and cannaboids that are of benefit in reducing the painful symptoms of SCD [126]. Additional phytomedicines that have been reported to possess antisickling potentials are *Pterocarpa osun*, *Justicia secunda,* and *Sorghum bicolor* extracts [127,128].

Taken together, these functional foods can be used in the form of food or decoction. The consumption of a cocktail of functional foods is highly recommended for sickle cell disease patients. Consuming functional foods and nutraceuticals alongside with/without FDA-approved drugs can be one of the first–line and promising therapies in managing the crises of SCD in Nigerian children especially during their infanthood stages.

## 6 Conclusion

For SCD challenging children in Nigeria, the functional food research and development must be motivated on how to decrease the number of crises during SCD and thus increase the overall survival of the SCD children. Various bioactive ingredients in the foods and edible plants may be extracted and screened to drug prologue during management of SCD crisis. Research to experimentally identify these bioactive ingredients is at hand. Therefore, capability of functional foods in managing SCD without painful episodes in children involves myriad of research into foods and nutraceuticals which is a current trend in nutritional biochemistry and genetic disorders like sickle cell disease, with a response to find readily available, cheaper and alternative novel molecule that the Nigerian children susceptible to SCD can get within their reach. This review paper indicates the therapeutic, antisickling, antioxidant and inhibitory effects of functional foods as combatants for sickle cell disease crises in Nigerian children (Table 2).

## Declarations

### Author contribution statement

All authors listed have significantly contributed to the development and the writing of this article.

### Funding statement

This research did not receive any specific grant from funding agencies in the public, commercial, or not-for-profit sectors.

### Data availability statement

No data was used for the research described in the article.

### Declaration of interests statement

The authors declare no conflict of interest.

### Additional information

No additional information is available for this paper.
